# Demographic data, habits of use and personal impression of the first generation of users of virtual reality viewers in Spain

**DOI:** 10.1016/j.dib.2018.11.127

**Published:** 2018-11-29

**Authors:** Roberto Sánchez-Cabrero, Amelia Barrientos-Fernández, Amaya Arigita-García, Lidia Mañoso-Pacheco, Oscar Costa-Román

**Affiliations:** a Alfonso X el Sabio University, Spain; b Nebrija University, Spain

## Abstract

Taking into account that the first virtual reality viewers started to be marketed in Spain at the end of 2016 (Gadelha, 2018; Parong and Mayer, 2018; Rizzo and Koenig, 2017) [1], [2], [3], a questionnaire was designed in order to show the social and demographic profile of this first generation of users of virtual reality experiences, itemising their ages, genders, educational level, professional field and present work status. Furthermore, the participants’ habits of use, interests, attitudes, assessments about the future potential of virtual reality in a range of areas and their preferences in this area are shown through the other items of the questionnaire.

A wide sample of 117 participants, who were early adopters of virtual reality viewers, was obtained posting a new thread in the virtual reality forum of the Internet website ‘*Elotrolado.net’.* The data were collected by means of an online questionnaire hosted at the private servers of ‘*Encuestafacil.com’.*

The sample did not undergo any pre-treatment and the obtained data were not altered.

**Specifications table**

**Table:** 

Subject area	*Social Sciences*
More specific subject area	*Social Sciences (General), Education, Human Factors and Ergonomics, Sociology and Political Science, Psychology (General)*
Type of data	*Questionnaire, text file, table, graph, figure.*
How data was acquired	*Online questionnaire hosted at the private servers of Encuestafacil.com.*
Data format	*Raw*
Experimental factors	*There was no sample pre-treatment*
Experimental features	*Individual and anonymous administration of an online questionnaire*
Data source location	*Spain*
Data accessibility	*Data are included in this article*
Related research article	*Domingo, J.R. y Gates Bradley, E. (2017). Education Student Perceptions of Virtual Reality as a Learning Tool. Journal of Educational Technology Systems.*doi:10.1177/0047239517736873.

**Value of the data**

• The data show the social and demographic profile of the virtual reality users in Spain. This technology has been present in the market for less than 2 years, thus the data obtained are unpublished and unique within the scientific field.
• The data show the virtual reality habits of use of the early adopters, which provides valuable information about the usefulness and interest of virtual reality in the present society.
• The data show the interests and assessments of the selected sample, which helps to get to know the settlement and acceptance of this technology among their users.
• The data show the future potential assessment of virtual reality in a range of fields through their users criteria, which could help to establish the settlement future evolution of this technology during the coming years.
• It is possible to analyse the contingencies and correlations between the shown data through the definition of the virtual reality present user profile, to assess their interests and to establish comparisons with other populations or with the use of different new technologies.

## 1 Data

The data herein produced belong to the administration of an anonymous questionnaire designed to assess the demographic profile, the habits of use and the personal impression of the first generation of users of virtual reality viewers in Spain.

A total of 578 questionnaires were administered and a final sample of 117 participants who completed the whole questionnaire was obtained, rejecting 36 participants who did not complete every question.

The data are presented in*.csv* format and were originally collected using the statistical software SPSS in.*sav* format, thanks to which all the variables could be correctly established in order to be analysed at a later stage.

The data have not being processed and are presented in a raw format, just as they have been previously collected from the online questionnaire designed *ad hoc* for this study.

**Fig. 1:**
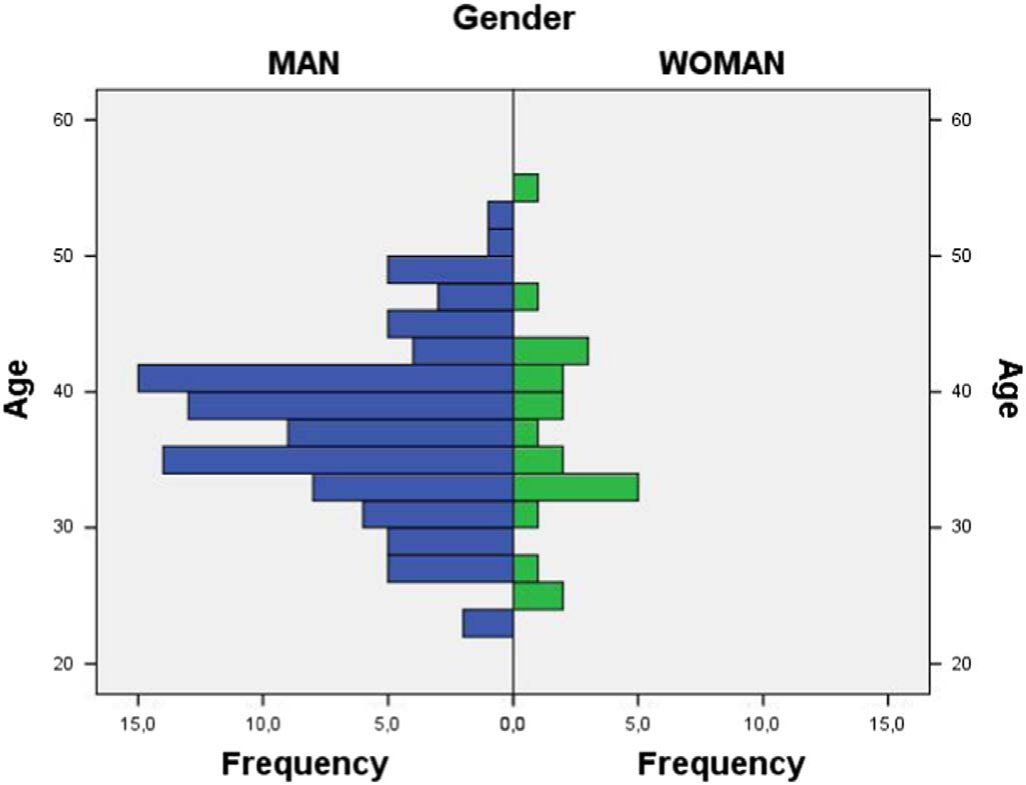
Age and gender pyramid.

**Fig. 2:**
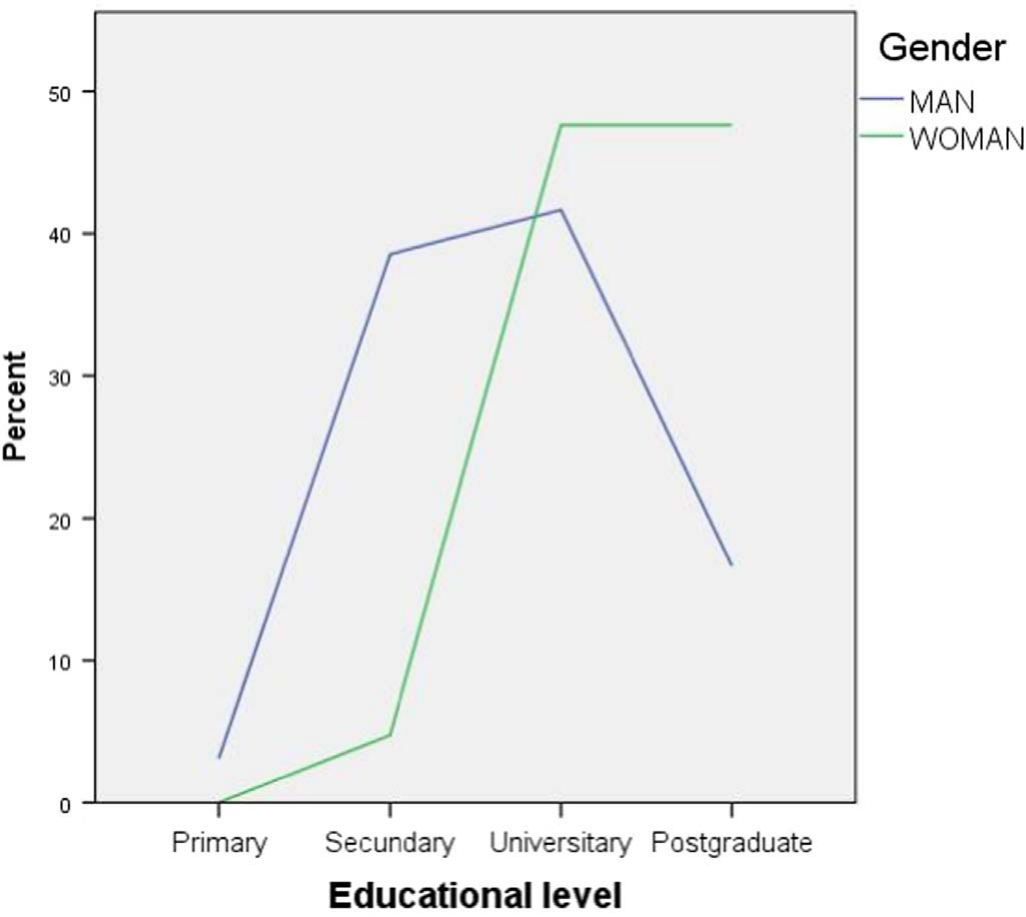
Educational level of the participants depending on gender.

**Fig. 3:**
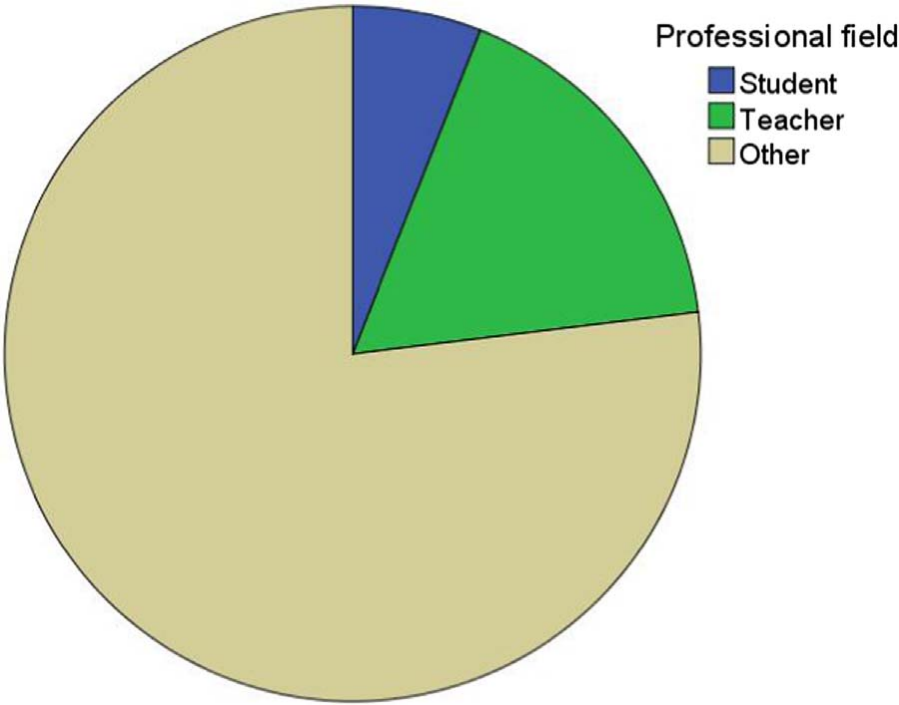
Professional field distribution of the sample (student, teacher, other).

**Fig. 4:**
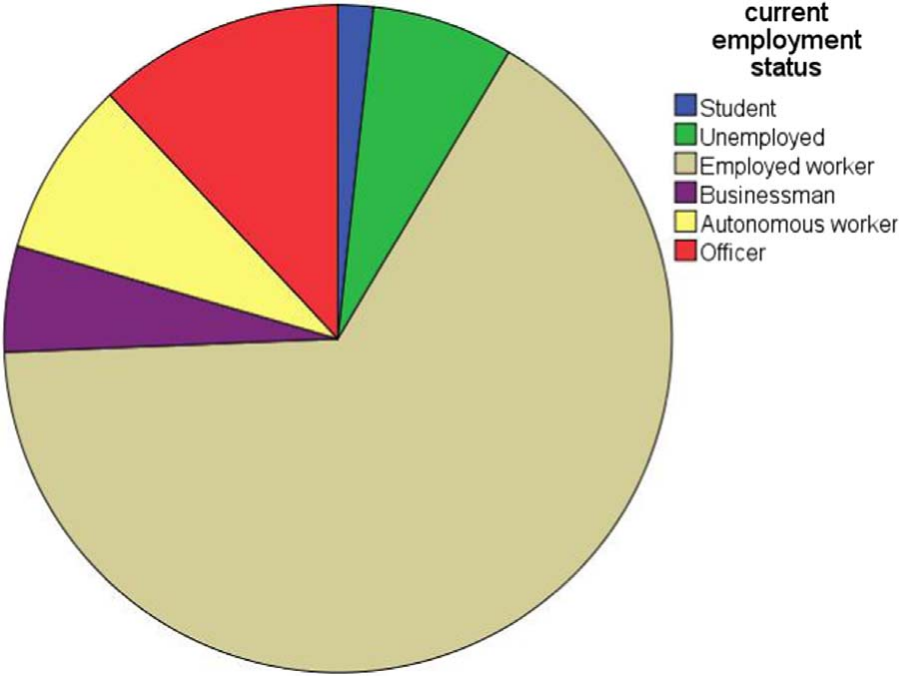
Current employment status distribution of the sample.

**Fig. 5:**
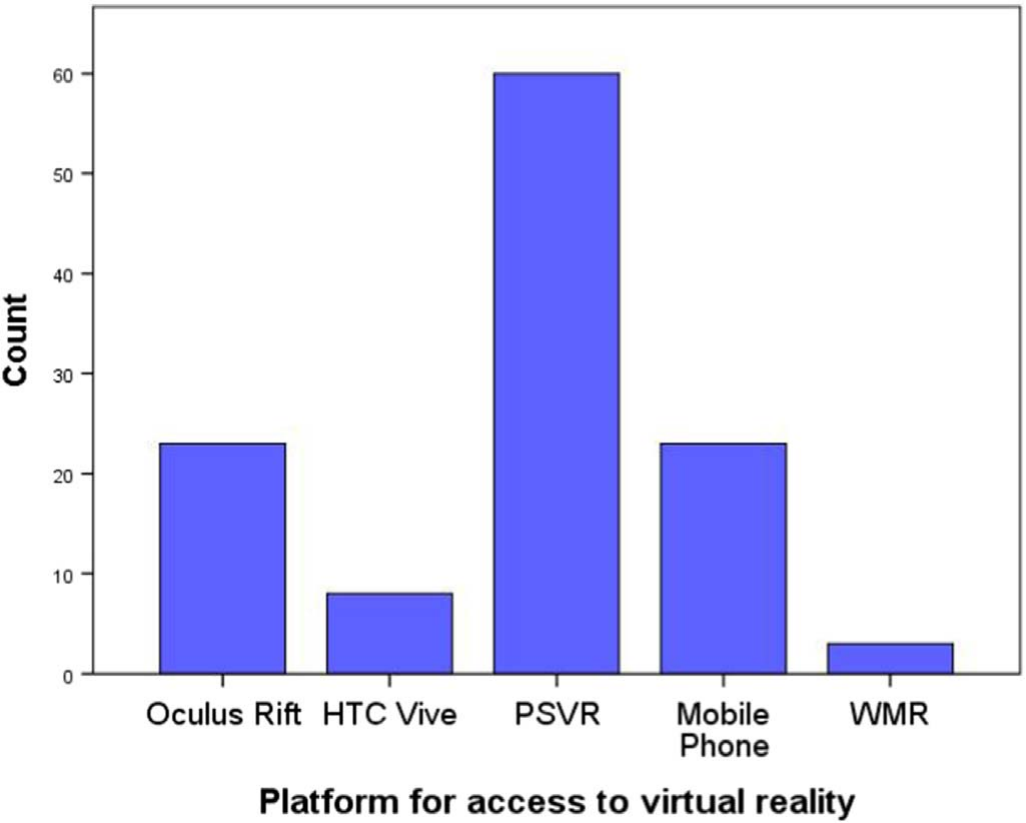
Platform for access to virtual reality distribution of the sample.

**Fig. 6:**
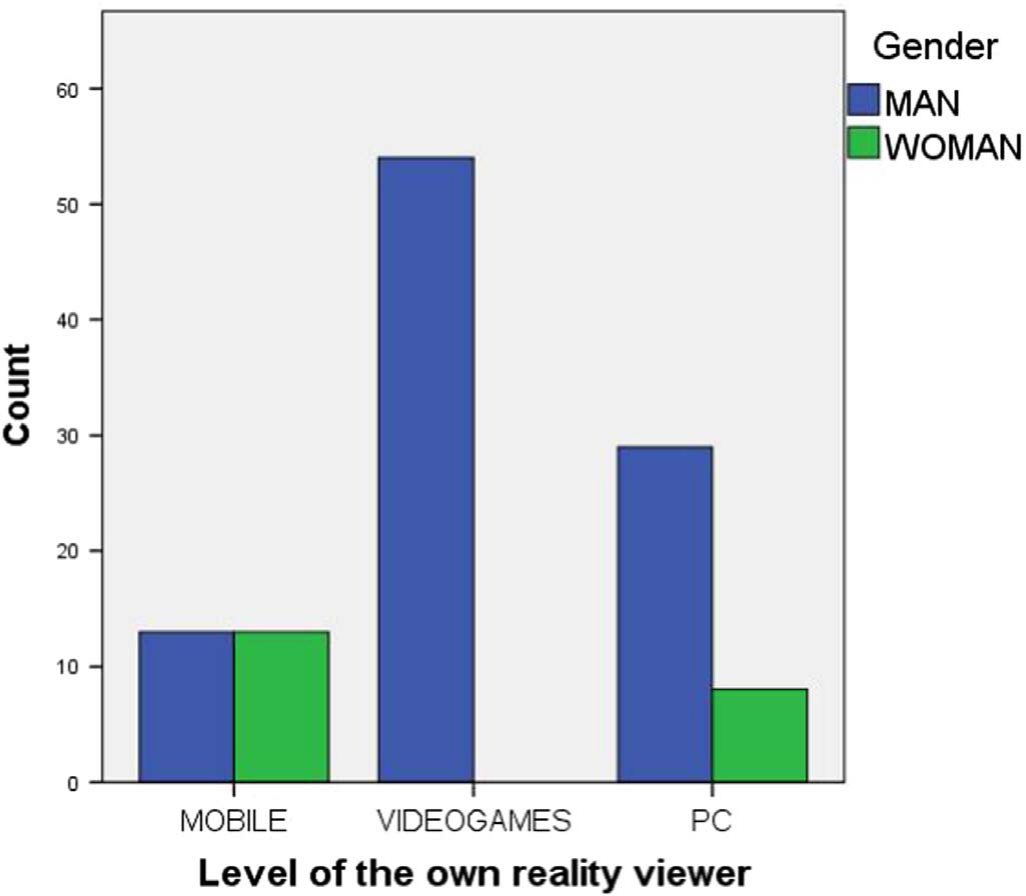
Level of the virtual reality viewer depending on the gender distribution of the sample.

The questionnaire shows the demographic (see Fig. 1) and social profile (see Fig. 2, Fig. 3, Fig. 4, Fig. 5, Fig. 6 as an example) of the virtual reality users in Spain by assessing: their age (discrete quantitative variable), their gender (man or woman), their educational level (primary, secondary, university, postgraduate) [1], [2], their professional field (student, teacher, others), their current work status (6 predefined categories), the level of the viewer they own (mobile phone, video game console or personal computer), the platform used to access virtual reality [3], platforms tried and favourite virtual reality platform (Oculus Rift, HTC Vive, PSVR, WMR and mobile phone platforms) [4].

**Fig. 7:**
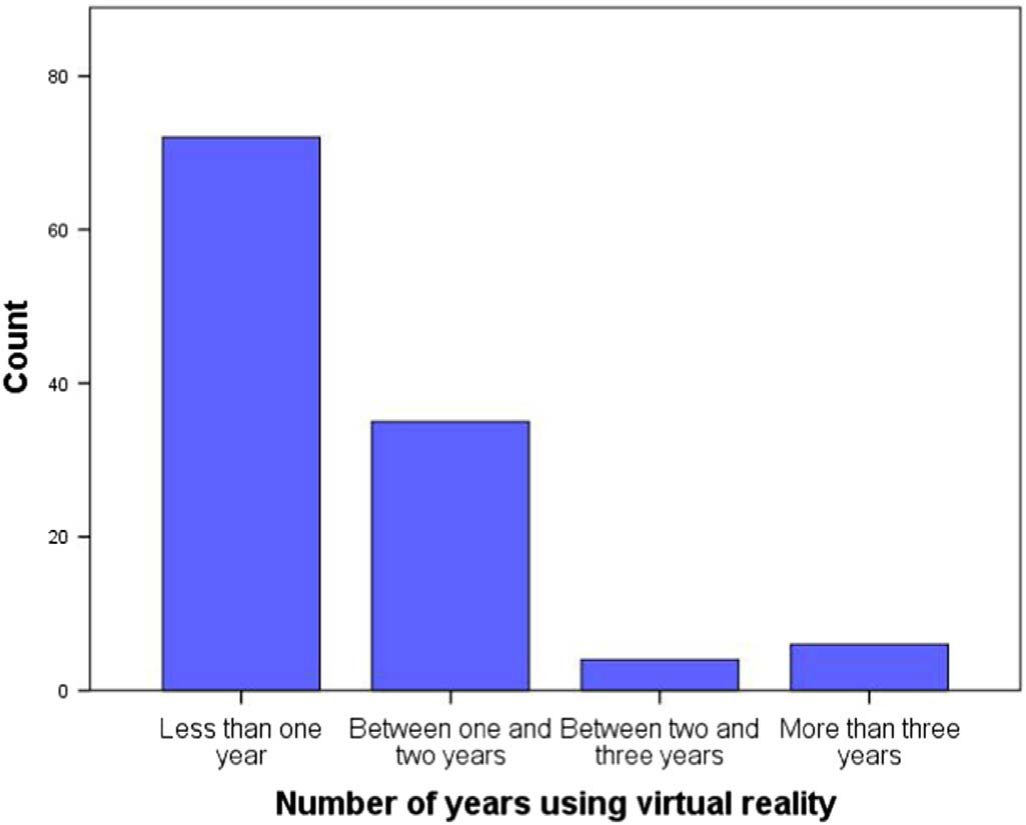
Number of years using virtual reality distribution of the sample.

**Fig. 8:**
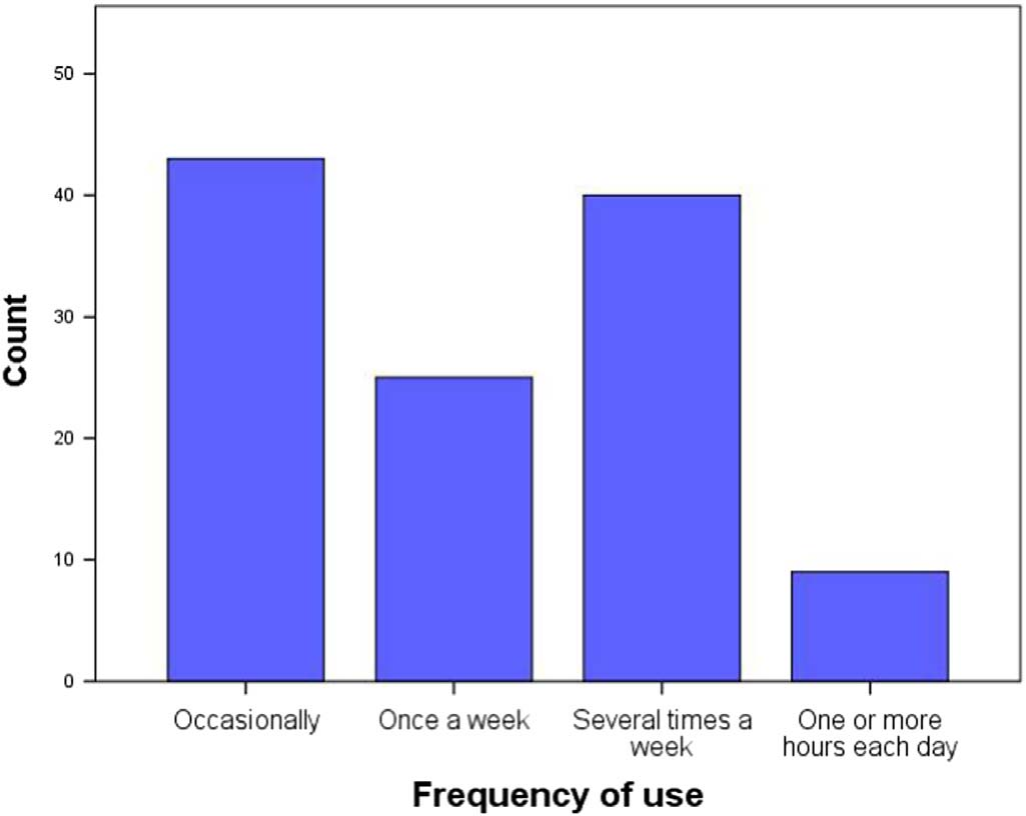
Frequency of use of virtual reality distribution of the sample.

Information about habits and frequency of use concerning virtual reality was requested (see Figs. 7 and 8 as an example) through the following items: how long they have owned a virtual reality viewer (5 predefined categories), Frequency of use of virtual reality (4 predefined categories of frequency), fields of use of virtual reality (7 predefined fields) and leisure genres of interest in virtual reality (14 predefined eligible genres plus the possibility of an open answer) [5], [6].

**Fig. 9:**
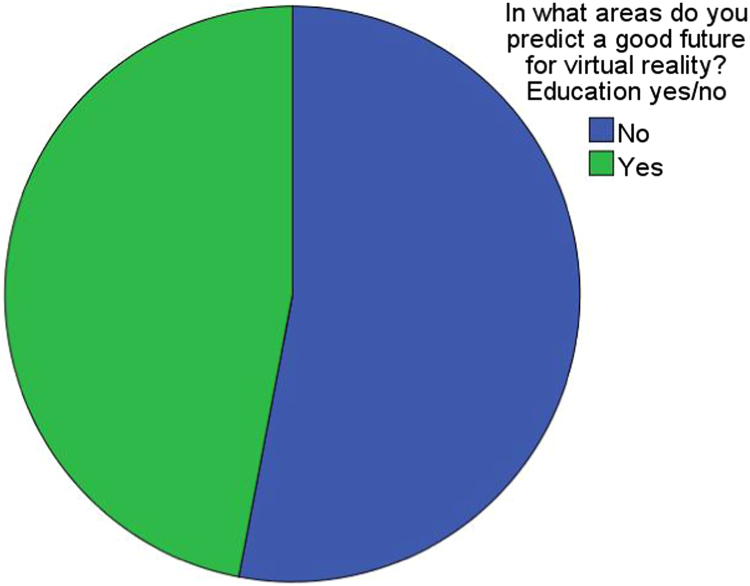
Percentage of the sample declared optimistic about the pedagogical future of virtual reality.

Finally, information about interests and assessments about the future of virtual reality was also requested (see Fig. 9 as an example) through the following items: Fields with an interest in the use of virtual reality in the future (7 predefined fields plus the possibility of an open answer), Fields with a certain future for virtual reality (9 predefined fields plus the possibility of an open answer), Restricting aspects which might prevent virtual reality from expanding in the present society (6 predefined restricting aspects plus the possibility of an open answer) [7], [8].

## 2 Experimental design, materials, and methods

An *ad hoc* online questionnaire was designed and hosted at the ‘*Encuestafacil.com’* private server, so that the participants could remotely access it from any kind of electronic device with Internet access.

The questionnaire and the protocol was assessed by the ‘*Scientific and Ethical Committee of the Nebrija University’* and went through a severe validation process led by external experts. Accepted written informed consent was obtained from all the participants in accordance with the Declaration of Helsinki (2013). The participants did not undergo any previous pre-treatment.

In order to achieve a sample of 117 users of virtual reality viewers marketed in Spain, a new thread in the virtual reality forum of the Internet website ‘*Elotrolado.net’* was posted, which is the most important Spanish speaking videogames and new technologies Internet website of the world. Participants accessed the questionnaire by using the hyperlink posted at the thread created in the virtual reality forum.

The questionnaire consisted of 6 pages and 30 questions, including dichotomous questions, with an only suggested option, with various suggested options and open questions. The distribution of contents was the following:

• Page 1: Compulsory acceptance of the written informed consent of the participant.
• Page 2: Demographic and social data of the participants in the study.
• Page 3: Previous experience description and frequency of use concerning the virtual reality viewers.
• Page 4: Interests and opinions description about virtual reality.
• Page 5: Assessments and qualitative opinions about the future of the technology.
• Page 6: Optional contact data requested in order to contact in the future.

Data shown in this study only include the answers obtained for questions from pages 2 to 4, as the answers collected from page 1 do not give any relevant information, the answers from page 5 include qualitative judgments without an assessable value and those from page 6 consist of personal data which must be protected in order to guarantee anonymity and privacy of the participants.

## References

[1] Gadelha R. (2018). Revolutionizing education: the promise of virtual reality. Child. Educ..

[2] Parong J., Mayer R.E. (2018). Learning science in immersive virtual reality. J. Educ. Psychol..

[3] Rizzo A.S., Koenig S.T. (2017). Is clinical virtual reality ready for primetime?. Neuropsychology..

[4] Moro C., Štromberga Z., Stirling A. (2017). Virtualisation devices for student learning: comparison between desktop-based (Oculus Rift) and mobile-based (Gear VR) virtual reality in medical and health science education. Australas. J. Educ. Technol..

[5] Buń P.K., Wichniarek R., Górski F., Grajewski D., Zawadzki P., Hamrol A. (2017). Possibilities and determinants of using low-cost devices in virtual education applications. EURASIA J. Math. Sci. Technol. Educ..

[6] Domingo J.R., Bradley E.G. (2018). Education student perceptions of virtual reality as a learning tool. J. Educ. Technol. Syst..

[7] Kim H., Ke F., Paek I. (2017). Game-based learning in an open sim-supported virtual environment on perceived motivational quality of learning. Technol. Pedagog. Educ..

[8] Nissim Y., Weissblueth E. (2017). Virtual Reality (VR) as a source for self-efficacy in teacher training. Int. Educ. Stud..

